# Supplementary figures and images for: Systemic degradation of repressive transcription factors gates gene expression and cell fate specification
Source: bioRxiv. 2026 Jun 18:2026.06.16.732780. Preprint. [Version 1] doi: 10.64898/2026.06.16.732780 (PMC13308096; doi:10.64898/2026.06.16.732780)

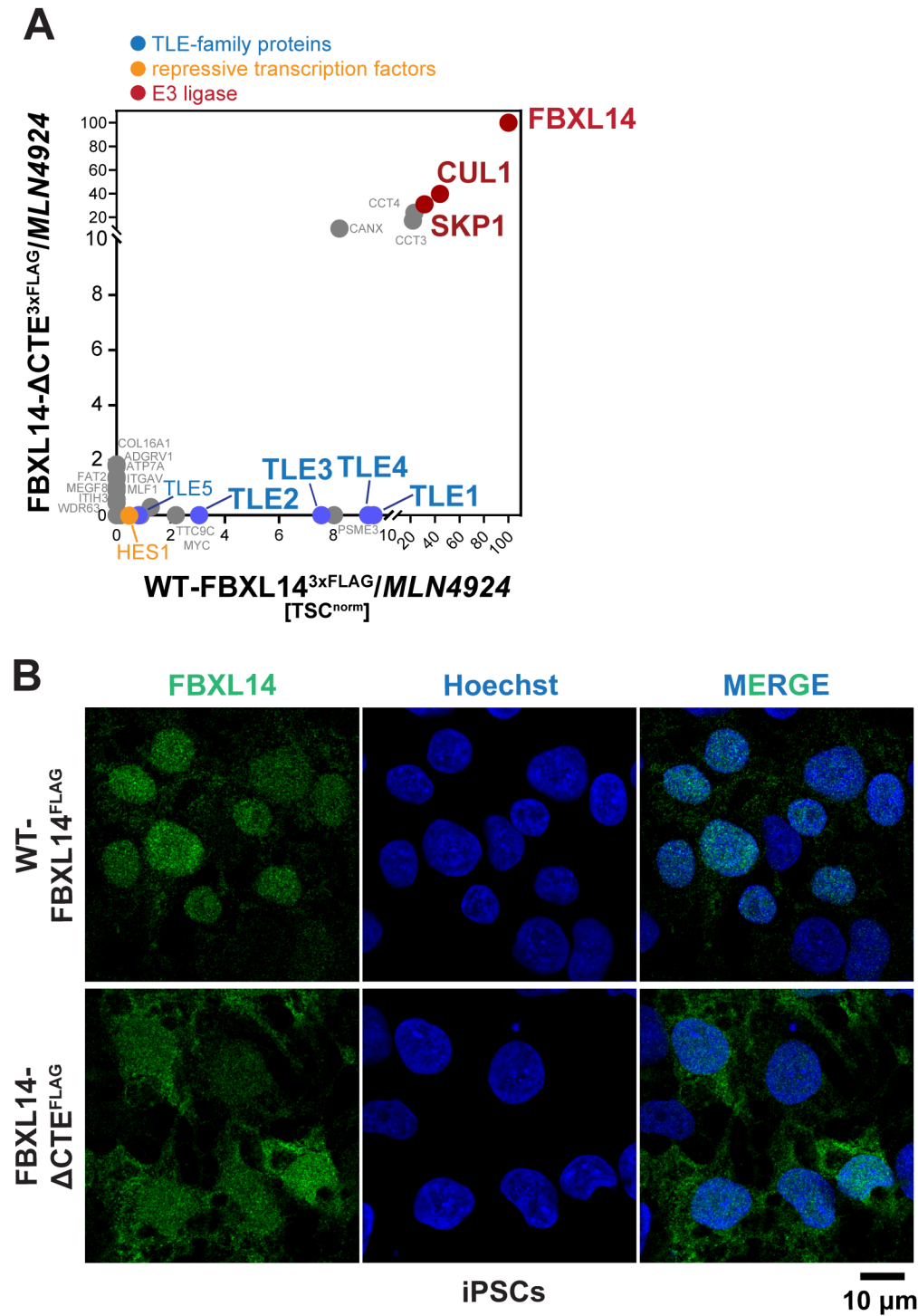

Figure S1

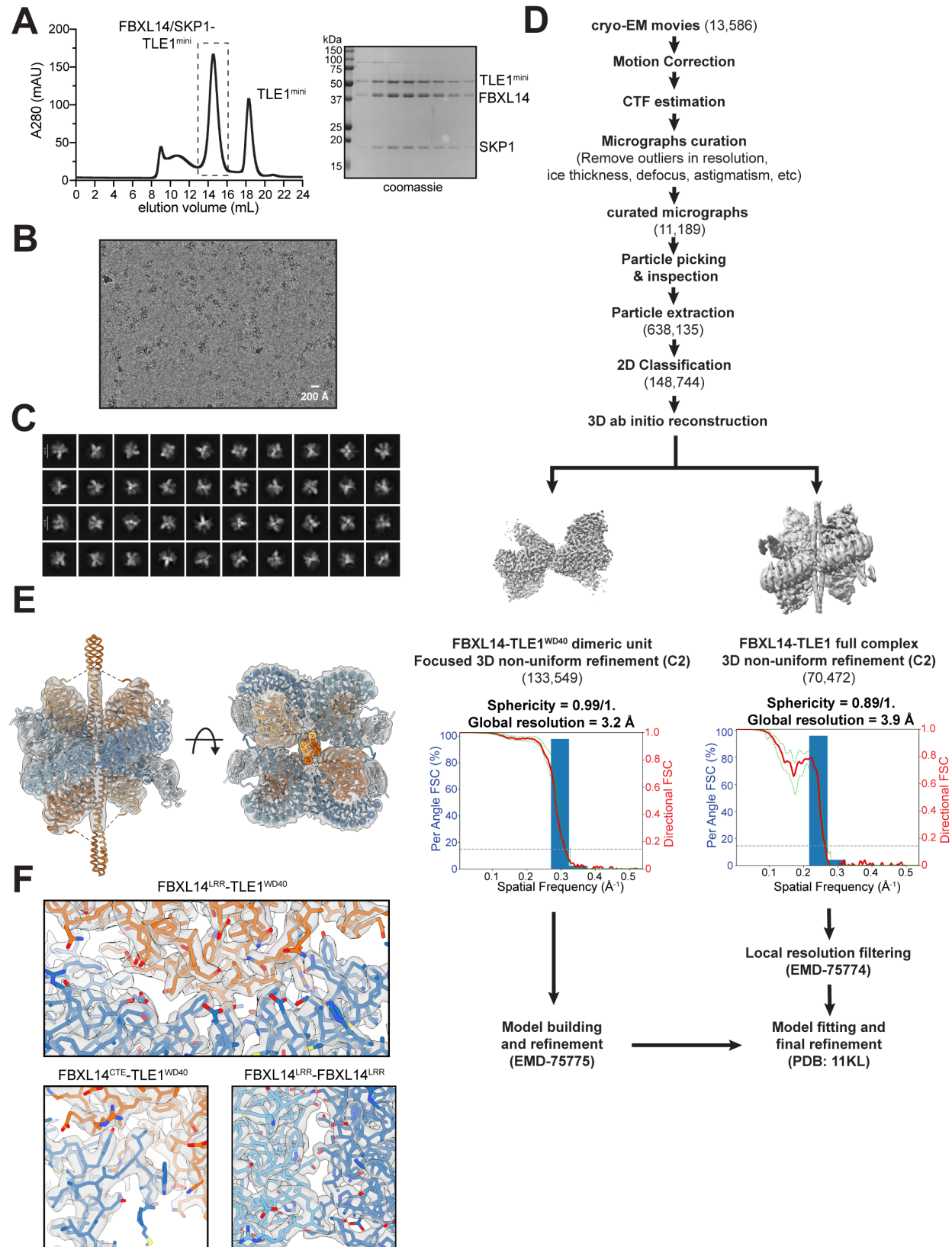

**Figure S2**

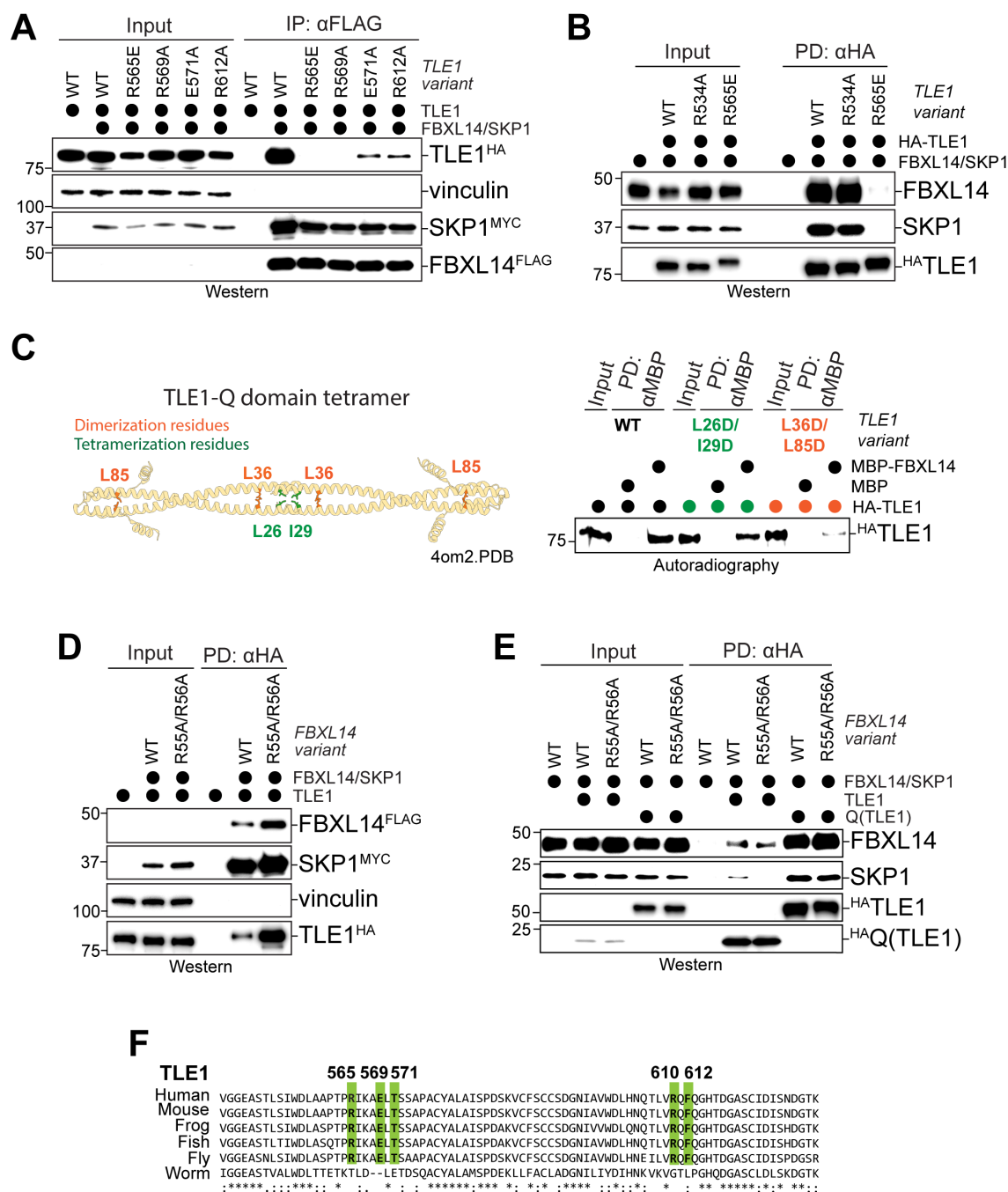

Figure S3

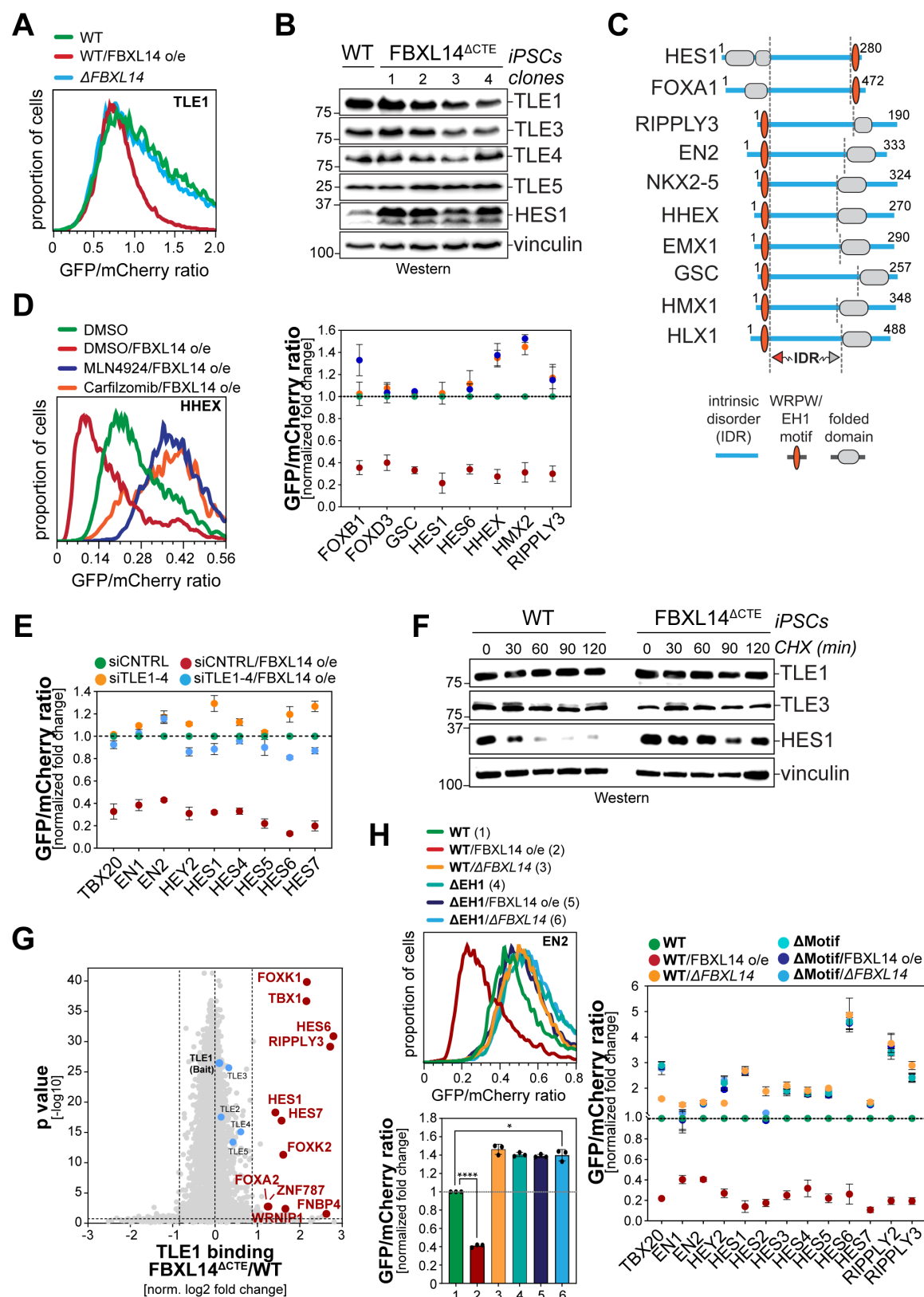

Figure S4

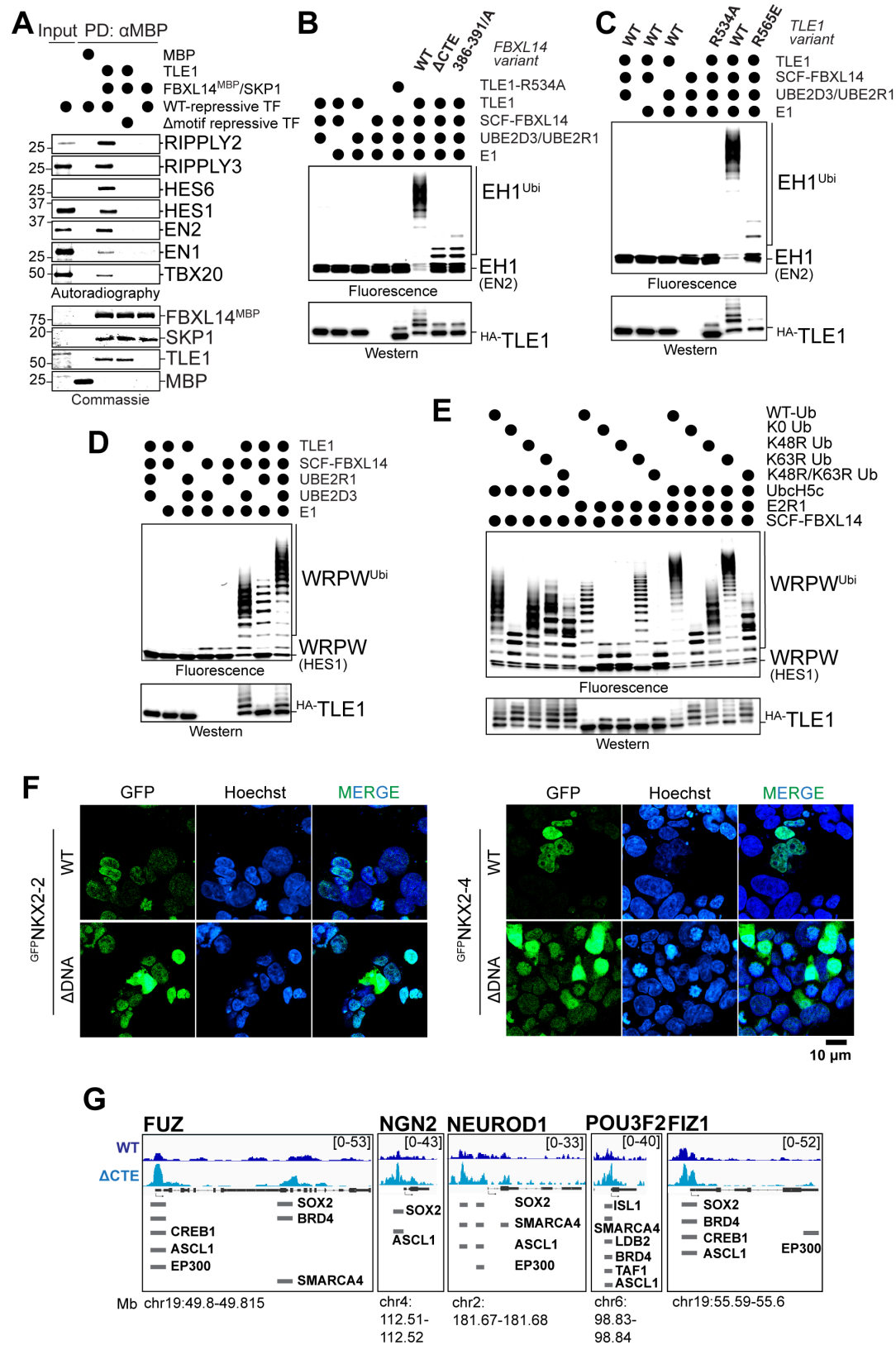

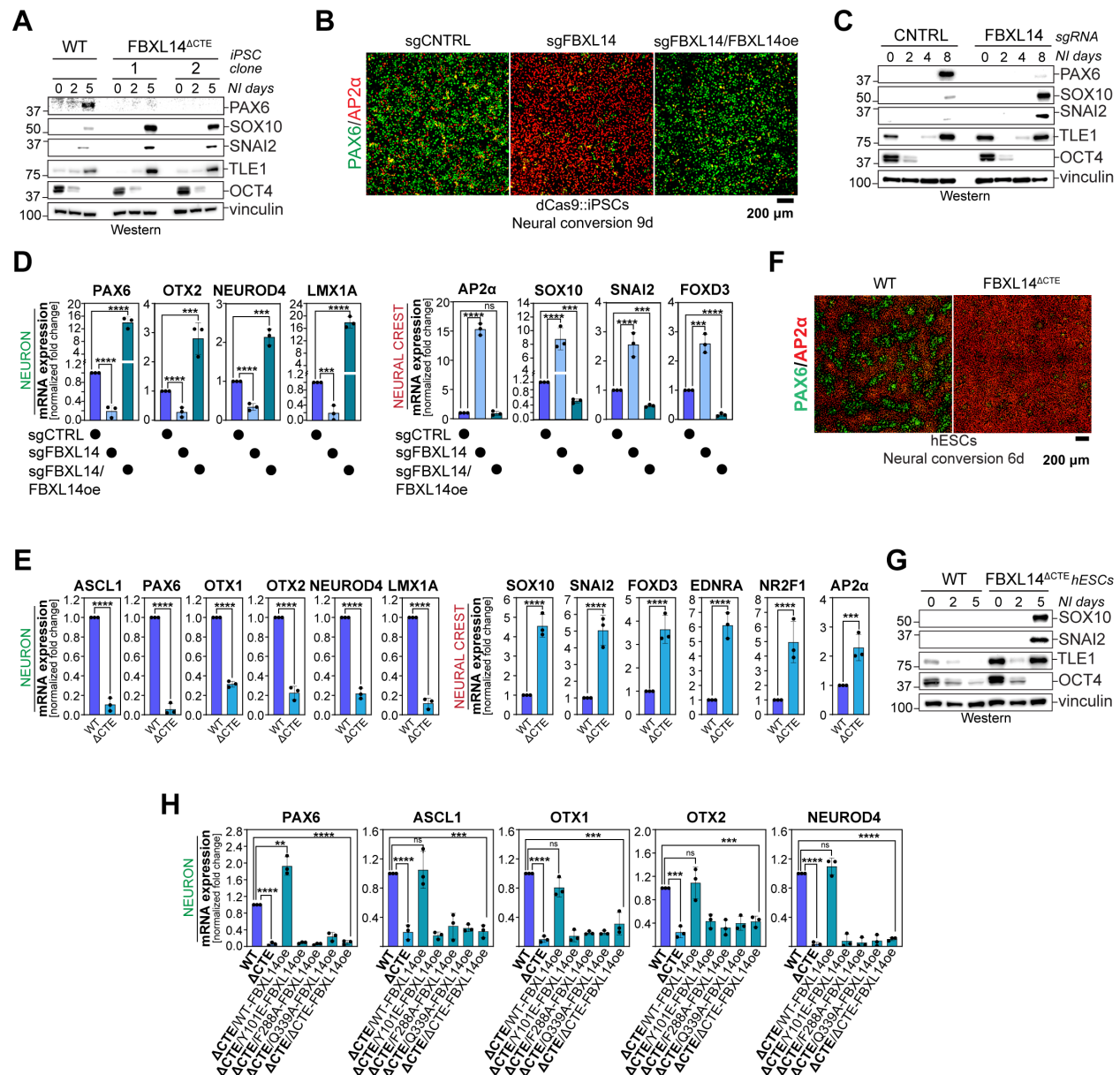

Figure S6

Supplement: Supplement 1 — Figure S1: The SCF substrate adaptor FBXL14 engages all TLE co-repressors. A. FBXL14 binds all members of the TLE family of co-repressors dependent on its CTE. Full-length FBXL143xFLAG or FBXL14ΔCTE/3xFLAG were affinity-purified from lysates of cells treated with the NEDD8-E1 inhibitor MLN4924 and binding partners were determined by mass spectrometry. B. FBXL14 localizes to the nucleus dependent on its interaction with TLEs. Localization of stably expressed FBXL143xFLAG or FBXL14ΔCTE/3xFLAG was determined by αFLAG immunofluorescence microscopy (green: FBXL14; blue: Hoechst). Figure S2: Cryo-EM structure of the mini-TLE1/FBXL14/SKP1 complex. A. Size exclusion chromatography trace and Coomassie-stained gel of FBXL14/SKP1/mini-TLE1 complex used for cryo-EM analysis. B. Motion-corrected and denoised representative micrograph. C. Representative 2D-class averages. D. Cryo-EM data processing particle flow and map resolution estimates. All processing steps were performed in CryoSparc. E. Full FBXL14/SKP1/mini-TLE1 model fit into the main cryo-EM map (EMD-75774) F. Model fit into the FBXL14/SKP1/TLE1WD40 high-resolution map obtained from a focused refinement of the region (EMD-75775). Figure S3: Validation of cryo-EM structure of the complex between FBXL14 and mini-TLE1. A. Mutations in TLE1 WD40-domain at the interface with the FBXL14 LRR-domain disrupt complex formation in cells. WT or mutant TLE13HA was co-expressed with FBXL143xFLAG/SKP13xMYC. FBXL14 was affinity-purified through αFLAG agarose, and co-precipitated TLE1 was detected by Western blotting. B. Mutations in the TLE1 WD40 disrupt binding to FBXL14 in vitro. WT- or mutant HATLE1 were immobilized and incubated with recombinant FBXL14/SKP1 complexes. Binding was visualized after gel electrophoresis by Western blotting. TLE1R534A is defective in transcription factor binding, while TLE1R565E has a mutation at the interface with FBXL14. C. Mutations in the TLE1 Q-domain disrupt binding to FBXL14 in vitro. MBP or MBPF [file NIHPP2026.06.16.732780v1-supplement-1.pdf]
